# Selective Ethylene Glycol Oxidation to Formate on Nickel Selenide with Simultaneous Evolution of Hydrogen

**DOI:** 10.1002/advs.202300841

**Published:** 2023-03-22

**Authors:** Junshan Li, Luming Li, Xingyu Ma, Xu Han, Congcong Xing, Xueqiang Qi, Ren He, Jordi Arbiol, Huiyan Pan, Jun Zhao, Jie Deng, Yu Zhang, Yaoyue Yang, Andreu Cabot

**Affiliations:** ^1^ Institute for Advanced Study Chengdu University Chengdu 610106 China; ^2^ College of Food and Biological Engineering Chengdu University Chengdu 610106 China; ^3^ School of Chemistry and Environment Southwest Minzu University Chengdu 610041 China; ^4^ Catalan Institute of Nanoscience and Nanotechnology (ICN2) CSIC and BIST Campus UAB, Bellaterra Barcelona Catalonia 08193 Spain; ^5^ Catalonia Institute for Energy Research—IREC Sant Adrià de Besòs Barcelona Catalonia 08930 Spain; ^6^ ICREA Pg. Lluís Companys 23 Barcelona Catalonia 08910 Spain; ^7^ School of Biological and Chemical Engineering Nanyang Institute of Science and Technology Nanyang 473004 China; ^8^ Hebei Key Laboratory of Photoelectric Control on Surface and Interface College of Science Hebei University of Science and Technology Shijiazhuang 050018 China; ^9^ Department of Materials Science and Engineering Pennsylvania State University University Park PA 16802 USA

**Keywords:** electrocatalysis, ethylene glycol electro‐oxidation, formate, hydrogen, nickel selenide, phase engineering

## Abstract

There is an urgent need for cost‐effective strategies to produce hydrogen from renewable net‐zero carbon sources using renewable energies. In this context, the electrochemical hydrogen evolution reaction can be boosted by replacing the oxygen evolution reaction with the oxidation of small organic molecules, such as ethylene glycol (EG). EG is a particularly interesting organic liquid with two hydroxyl groups that can be transformed into a variety of C1 and C2 chemicals, depending on the catalyst and reaction conditions. Here, a catalyst is demonstrated for the selective EG oxidation reaction (EGOR) to formate on nickel selenide. The catalyst nanoparticle (NP) morphology and crystallographic phase are tuned to maximize its performance. The optimized NiS electrocatalyst requires just 1.395 V to drive a current density of 50 mA cm^−2^ in 1 m potassium hydroxide (KOH) and 1 m EG. A combination of in situ electrochemical infrared absorption spectroscopy (IRAS) to monitor the electrocatalytic process and ex situ analysis of the electrolyte composition shows the main EGOR product is formate, with a Faradaic efficiency above 80%. Additionally, C2 chemicals such as glycolate and oxalate are detected and quantified as minor products. Density functional theory (DFT) calculations of the reaction process show the glycol‐to‐oxalate pathway to be favored via the glycolate formation, where the C—C bond is broken and further electro‐oxidized to formate.

## Graphical abstract

A combination of in situ and ex situ analysis shows the main product of the ethylene glycol (EG) oxidation reaction (EGOR) is formate with a Faradaic efficiency above 80%, and glycolate and oxalate as minor chemicals on nickel selenide nanoparticles (NPs). Further density functional theory (DFT) calculation reveals the electrooxidation mechanism to these products.

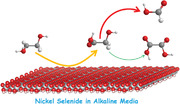

## 1 Introduction

Molecular hydrogen is both a fundamental precursor in the chemical industry and an extremely appealing, potentially carbon‐free, energy carrier.^[^ ^1^, ^2^ ^]^ However, H_2_ is currently being produced mainly from fossil fuels, releasing large amounts of carbon dioxide. The production of H_2_ from water electrolysis is not cost effective in front of the steam reforming or gasification of fossil fuels due to its moderate energy efficiency and the use of expensive noble metal catalysts.^[^ ^3^ ^]^ Thus alternative strategies for the production of hydrogen from net‐zero carbon sources and using renewable energies need to be developed.

In the electrochemical water splitting, most energy is consumed at the anode due to the sluggish oxygen evolution reaction (OER).^[^ ^3^, ^4^, ^5^, ^6^ ^]^ To overcome this limitation, OER can be replaced with the oxidation of small molecules having lower oxidation potentials, which implies higher energy efficiencies and the potential use of lower‐cost catalysts based on abundant elements.^[^ ^7^, ^8^, ^9^ ^]^ Thus, potentially, hydrogen can be cost‐effectively evolved from aqueous solutions containing biomass‐derived products or organic waste such as ethanol, glycerol, ethylene glycol (EG), or sugars with a net‐zero CO_2_ footprint.^[^ ^10^, ^11^, ^12^ ^]^ An additional advantage of the production of hydrogen from the reforming of organic waste or biomass‐derived organics is the potential co‐generation of valuable organic chemicals, which can improve the process economics and eventually reduce the use of fossil resources to produce these chemicals.^[^ ^13^, ^14^, ^15^ ^]^ As an example in this last direction, in previous studies we demonstrated that formate, a key precursor in the chemical and pharmaceutical industries, can be electrochemically produced from methanol in alkaline media using nickel catalysts, which can be economically advantageous over current methods based on high temperature and pressure conditions.^[^ ^16^, ^17^, ^18^ ^]^ Compared with methanol, EG is a nontoxic liquid with high boiling point and low volatility, which offers safe storage and high volumetric energy density. In addition, EG can be renewably obtained through efficient biomass‐to‐ethylene glycol processes.^[^ ^19^ ^]^ Besides, the EG market price is slightly below that of formic acid, thus the production of hydrogen from EG reforming to formate involves no loss of product value at the anode side.^[^ ^20^ ^]^

Recently, the electrocatalytic oxidation of glycols has been extensively studied, especially on noble‐metal‐based electrocatalysts.^[^ ^21^, ^22^, ^23^, ^24^, ^25^, ^26^, ^27^ ^]^ Si et al. reported the electro‐oxidation of glycolic acid with a very low cell potential on PdAg supported on nickel foam.^[^ ^25^ ^]^ In another recent study, Pd–PdSe heterostructures showed a high EG oxidation reaction (EGOR) selectivity, above 44%, to C1 products, with superior C—C bond cleavage.^[^ ^24^ ^]^ However, the use of noble metals hampers the industrial application of these strategies.

Nickel is an excellent alternative catalyst to noble metals for the electro‐oxidation of small molecules in alkaline media. In a basic solution, the surface of nickel is oxidized to nickel oxyhydroxide, NiOOH, which is the active surface for organic electro‐oxidation.^[^ ^28^, ^29^, ^30^, ^31^ ^]^ On a typical Ni‐based catalyst, small organics containing only one hydroxyl can be electrochemically transformed into their corresponding acidic commodities, such as methanol to formate and ethanol to acetate with high Faradaic efficiency.^[^ ^17^, ^32^, ^33^ ^]^ EG, functionalized with two hydroxyls, displays different routes for the transformation to C1 and C2 products. Specifically, the EGOR primarily involves two pathways, either a 10‐electron process to direct/complete oxidation that results in carbonate (in an alkaline environment), or a multielectron process resulting in indirect/incomplete oxidation into C1 or C2 chemicals, including glyoxal, glycolaldehyde, glycolate, glyoxalate, oxalate, and formate.^[^ ^34^ ^]^

Nickel compounds, such as nitrides, phosphides, and chalcogenides, have frequently demonstrated promoted electrocatalytic performance over bare nickel.^[^ ^35^ ^]^ We and others have demonstrated the surface of such compounds to be reconstructed to more active hydroxide and mainly oxohydroxide, which are recognized as the true catalytically active surfaces for OER in alkaline media.^[^ ^35^, ^36^, ^37^ ^]^ During this surface reconstruction, the surface anions are simultaneously oxidized to nitrates (NO_3_^−^), phosphates (PO_4_^3−^), sulfates (SO_4_^2−^), and selenates (SeO_4_^2−^).^[^ ^38^, ^39^, ^40^ ^]^ Such anionic species can modulate the surface electronic structure improving the material catalytic performance.^[^ ^41^ ^]^ As an example, Li et al. studied the influence of different oxyanions obtained from Ni metalloids (NiT*_x_*, T = P, S, and Se) on the coordination environments of Ni sites to optimize the methanol electro‐oxidation performance.^[^ ^42^ ^]^ Nevertheless, to the best of our knowledge, the electro‐oxidation mechanism of glycols on nickel surfaces is yet to be explored.

Herein, we prepared a series of nanostructured nickel diselenides with tuned crystal phase and morphology from a simple single‐precursor ink. The different nanostructures are tested toward the electrocatalytic EGOR. In situ infrared absorption spectroscopy (IRAS), ex situ analyses of the electrolyte and materials, and density functional theory (DFT) calculations are used to analyze the reaction product and gain insight into the process, the active sites, and the influence of the different components.

## 2 Results and Discussion

### 2.1 Nanomaterials’ Synthesis and Characterization

**Figure 1:**
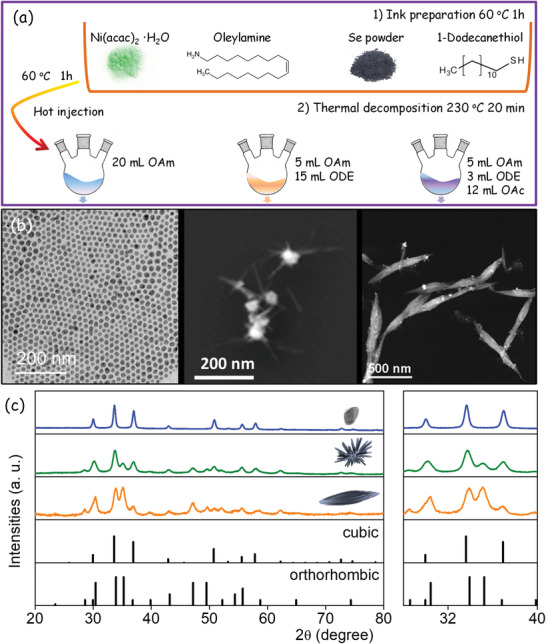
a) Scheme of the two‐step process used to produce nanostructured nickel selenide. b) TEM images. c) XRD patterns of the obtained materials. The reference patterns of the cubic (JCPDS 00‐041‐1495) and orthorhombic (JCPDS 00‐018‐0886) NiSe_2_ phases are also plotted.

NiSe_2_ nanostructures were produced in two steps (**Figure** **1**a). In the first step, a single Ni–Se precursor was prepared by dissolving Ni acetylacetonate and selenium (Se) powder in a solution containing oleylamine (OAm, C_18_H_37_N) and 1‐dodecanethiol (DDT, CH_3_(CH_2_)_11_SH). In the second step, this ink was thermally decomposed in the presence of a mixture of solvents/surfactants (OAm/1‐Octadecene (ODE)/oleic acid (OAc)) that determined the final particle shape and crystallographic phase (see the “Experimental Section” for details). Figure 1b,c shows representative transmission electron microscopy (TEM) images and the X‐ray diffraction (XRD) patterns of the materials produced using different OAm/OAc/ODE combinations. In the presence of just OAm, quasispherical nanoparticles (NPs) with a narrow size distribution centered at ≈15 nm and pure cubic NiSe_2_ phase (JCPDS 00‐041‐1495) were obtained. When adding ODE into the OAm solution (*V*_OAm_:*V*_ODE_ = 1:3), ≈200 nm branched nanostructures with 50–100 nm arms and mixed cubic and orthorhombic phases were produced. Besides, when OAc was added into the solution (*V*_OAm_:*V*_ODE_:*V*_OAc_ = 5:3:12), elongated bundles with a diameter of about 100 nm, a length of ≈0.5–0.8 µm and pure orthorhombic phase (JCPDS 00‐018‐0886) were obtained. As shown in Figure S1 (Supporting Information), the Ni:Se atomic ratio for these three structures was determined to be 1:2, in good agreement with stoichiometric NiSe_2_.

**Figure 2:**
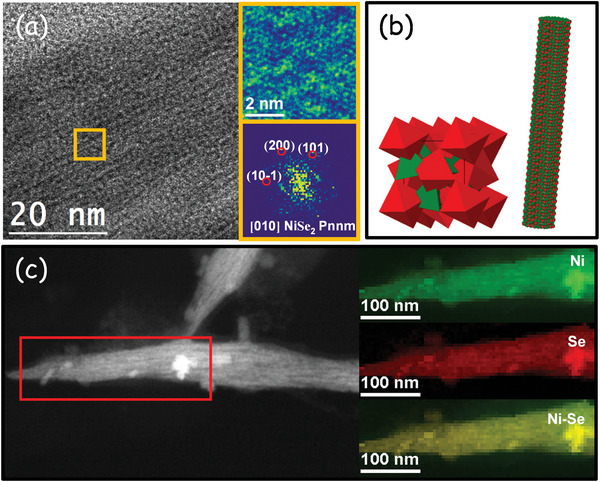
a) HRTEM image, detail of the orange squared region and its corresponding power spectrum of a NiSe_2_ bundle. From the crystalline domain, the lattice fringe distances were measured to be 0.295, 0.267, and 0.299 nm, at 53.99° and 108.96°, which can be interpreted as the cubic NiSe_2_ phase, visualized along its [010] zone axis. b) 1*1*1 unit crystal model of NiSe_2_ and 3D atomic supercell model of the NiSe_2_ bundles. Red and green particles represent Ni and Se, respectively. c) EELS chemical composition maps obtained from the red squared area of the STEM image.

**Figure 3:**
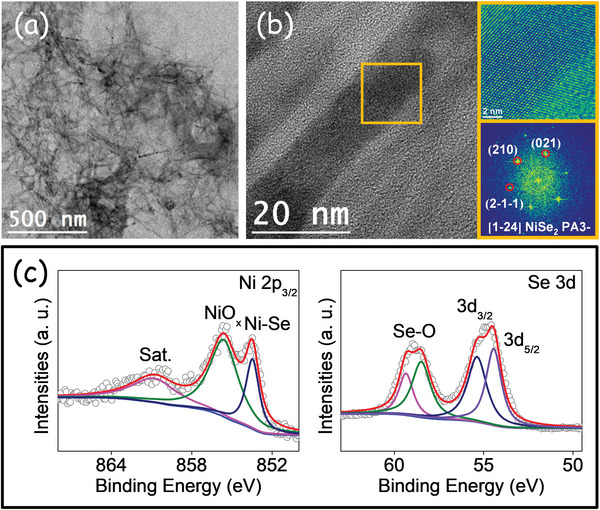
a) TEM image of branched NiSe_2_ NPs. b) HRTEM image of an NP branch, detail of the orange squared region and its corresponding power spectrum. c) Ni 2p_3/2_ and Se 3d high‐resolution XPS spectra.

As displayed in **Figure** **2**a and Figure S2a,b (Supporting Information), high‐resolution TEM (HRTEM) and electron diffraction analyses confirmed the presence of the orthorhombic (space group = *Pnnm*, *a* = 4.89 Å, *b* = 5.96 Å, and *c* = 3.67 Å) and cubic lattices (space group *PA3‐*, *a* = *b* = *c* = 5.9730 Å) in the NiSe_2_ bundles and spherical NPs, respectively. The crystal model of NiSe_2_ and an atomic supercell model are presented in Figure S2b,c (Supporting Information) for the spherical NPs and bundles, respectively. Electron energy loss spectroscopy (EELS) chemical composition maps show a homogeneous distribution of Ni and Se within each particle (Figure 2c; Figure S2d, Supporting Information). **Figure** **3**a shows the high‐angle annular dark‐field (HAADF)–scanning TEM (STEM) and HRTEM images of the branched structure. As can be seen in Figure 3b, the detail of the orange squared region and its corresponding power spectrum reveals the presence of the NiSe_2_ cubic lattice in this sample.

Within our system, OAm acts as a strong ligand, preventing the growth of the NiSe_2_ crystals and resulting in NPs with the thermodynamically stable cubic crystal structure and a quasispherical morphology associated with the high symmetry of the cubic crystallographic phase. When adding ODE as a solvent, i.e., reducing the OAm concentration, the action of the OAm as a capping agent is reduced; a faster growth rate is obtained; and the formation of some kinetically controlled orthorhombic NiSe_2_ takes place. Because the orthorhombic phase is highly anisotropic, the growth rate along the different crystallographic directions is not homogeneous. In particular, we observe the growth rate along the [001] direction to be much faster, which results in the growth of some elongated structures from the surface of the crystals.^[^ ^43^ ^]^ When adding significant amounts of OAc, there is a competition between the OAm and OAc binding to the surface and also with the formation of OAm–OAc complexes.^[^ ^44^ ^]^ With an excess amount of OAc, the growth rate is further accelerated and relatively large bundles with a pure orthorhombic crystal structure are formed. While the detailed growth mechanism is not completely understood, it is worth noting that the phase‐controlled synthesis reported herein bears some similarity to the synthesis of other metal oxide and chalcogenide NPs in the presence of OAc and OAm.^[^ ^44^ ^]^

X‐ray photoelectron spectroscopy (XPS) was used to study the surface chemistry of the produced nickel selenides (Figure 3c; Figures S3 and S4, Supporting Information). As shown in Figure 3c, the high‐resolution Ni 2p_3/2_ XPS spectrum for the branched sample displays three obvious peaks, including a shake‐up satellite (marked “Sat.”) at 860.9 eV. The peak located at 853.5 eV is assigned to Ni^2+^ within the selenide lattice.^[^ ^45^ ^]^ Besides, the peak at 855.7 eV is assigned to Ni^2+^ within a more electronegative chemical environment probably generated by the surface oxidation of the particles.^[^ ^46^ ^]^ The high‐resolution Se 3d XPS spectrum displays two doublets. The first doublet is located at 54.4 eV (Se 3d_5/2_) and it is assigned to Se^2−^ within the metal diselenide environment.^[^ ^47^ ^]^ The second doublet at 58.9 eV (Se 3d_3/2_) is assigned to an oxidized Se environment (SeO*_x_*) generated by the exposure of the particles to the air atmosphere.^[^ ^47^ ^]^ As can be seen in Figure S4 (Supporting Information), the XPS characteristics of the spherical NPs and bundles did not change with respect to the branched NiSe_2_ morphology.

### 2.2 Electrocatalytic Performance

**Figure 4:**
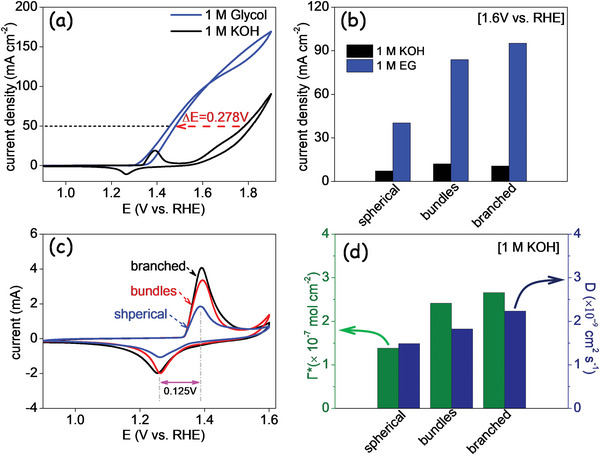
a) CV curve of an electrode based on branched NiSe_2_ particles in 1 m KOH with and without 1 m EG in the potential range of 0.9–1.9 V versus RHE. b) Current density of NiSe_2_‐based electrodes at 1.6 V versus RHE in 1 m KOH with and without 1 m EG. c) CV curves of NiSe_2_‐based electrode in 1 m KOH in the potential range of 0.9–1.6 V versus RHE. d) Surface coverage of active species (Γ*) and apparent diffusion coefficient (*D*) of the nanostructured nickel selenide electrodes.

NiSe_2_ nanostructures were supported on glassy carbon (GC) to characterize their electrocatalytic performance toward EGOR (see details in the “Experimental Section”). As shown in **Figure** **4**a and Figure S5 (Supporting Information), cyclic voltammetry (CV) in the potential range from 0.9 to 1.9 V versus reversible hydrogen electrode (RHE) was initially conducted in 1 m potassium hydroxide (KOH) at a scan rate of 50 mV s^−1^. In alkaline electrolyte, the anodic peak at 1.391 V measured in the forward sweep corresponds to the Ni(OH)_2_/NiOOH oxidation, and the cathodic peak at 1.266 V in the backward sweep is attributed to the NiOOH/Ni(OH)_2_ reduction (black curve).^[^ ^48^ ^]^ When adding 1 m EG into the electrolyte, a sharp rise in the current density was observed at ≈1.405 V (blue curve), pointing that NiOOH is recognized as the main active species for EG electro‐oxidation.^[^ ^49^ ^]^ Without EG, 1.595 V was required to drive a current density of 10 mA cm^−2^, while just 1.389 V was needed in the presence of 1 m EG. However, Pd and Pt catalysts are certainly characterized by much lower on‐set potentials, down to just 0.5–0.6 V versus RHE, which allows the manufacturing of direct EG fuel cells.^[^ ^21^, ^24^, ^25^ ^]^ For the case of noble‐metal‐free electrocatalysts, the onset voltages measured for NiSe_2_ in alkaline media in the presence of EG are much lower than that required to drive the OER, which could concurrently promote a more efficient H_2_ generation at the cathode.

Among the different NiSe_2_ samples, the branched particles displayed the lowest EGOR overpotentials (Figure S5, Supporting Information). Figure 3b compares the current densities obtained at 1.6 V from electrodes based on NiSe_2_ particles with different morphologies/phases. As can be seen, the branched nanostructures have the highest electrocatalytic performance, with 95.23 mA cm^−2^, well above the 40.25 mA cm^−2^ obtained from spherical NPs and the 83.95 mA cm^−2^ for bundles.

Figure 4c shows the CVs of the three electrodes in 1 m KOH solution at a scan rate of 50 mV s^−1^ in the potential window from 0.9 to 1.6 V. The potential difference of redox peaks (Δ*E*_p_), revealing the rate of the electron transfer kinetics, was 0.125 V for all the samples, regardless of their morphology and crystal phase.^[^ ^49^ ^]^

The surface coverage of active species (Γ*) and the apparent diffusion coefficient (*D*) were determined from CVs at different sweep rates, in the range from 10 to 100 mV s^−1^ (see Figure S6 and calculation details in the Supporting Information).^[^ ^50^, ^51^ ^]^ From the linear fitting of the dependence of the anodic and cathodic peak currents with the sweep rate in the range from 10 to 50 mV s^−1^, a Γ*= 2.7 × 10^−7^ mol cm^−2^ was calculated for the branched NiSe_2_‐based electrode (Figure 4d). This value decreased to Γ*= 2.4 × 10^−7^ mol cm^−2^ for bundles and Γ*= 1.4 × 10^−7^ mol cm^−2^ for spherical nanostructures. The peak current densities linearly change with the square root of the sweep rate in the range of 60–100 mV s^−1^, demonstrating the redox reaction to be diffusion limited. From the fitting, the apparent diffusion coefficient was *D* = 2.23 × 10^−9^ cm^2^ s^−1^ for branched NiSe_2_, which was slightly above that of bundles (*D* = 1.83 × 10^−9^ cm^2^ s^−1^) and spherical (*D* = 1.49 × 10^−9^ cm^2^ s^−1^) nanostructured electrodes (Figure 4d).

CVs at different sweep rates in the non‐Faradaic region allowed for estimating the electrochemical surface area (ECSA; Figure S7, Supporting Information).^[^ ^52^, ^53^ ^]^ ECSA was found to increase from 5.3 cm^−2^ for spherical NPs and 6.3 cm^−2^ for bundles, to 9.65 cm^−2^ for branched nanostructures. Besides, electrochemical impedance spectroscopy (EIS) showed a higher electrical conductivity and lower charge‐transfer resistance for the electrodes based on branched structures, compared with those based on spherical NPs or bundles (Figure S8, Supporting Information). Overall, the higher EGOR performance of branched particles was consistent with their higher surface coverage of active species, diffusion coefficient, ECSA, and charge‐transport properties provided by their particular geometry.

### 2.3 In Situ and Ex Situ Electrocatalytic Study

**Figure 5:**
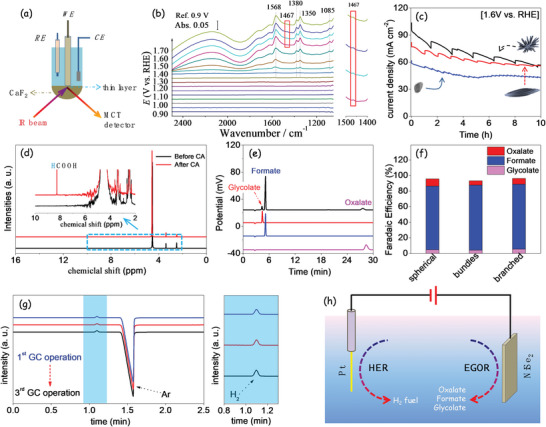
a) Schematic drawing of the IRAS set‐up. b) In situ IRAS spectra of EGOR on branched nickel selenide in 1 m KOH and 1 m EG in the potential range of 0.9–1.7 V versus RHE, taken the spectrum collected at 0.9 V vs. RHE as the reference spetrum. c) CA response for the three electrodes at 1.6 V with continuous 10 h operation in 1 m KOH and 1 m EG. d,e) Ex situ ^1^H NMR spectra and IC profiles for the electrolyte after CA measurement of the electrode based on branched nickel selenide. f) Faradaic efficiencies based on IC results. g) GC curves with three samplings at the cathode. h) Schematic drawing of the coupling hydrogen evolution reaction (HER) and EGOR.

Generally, IRAS was performed to study the electrocatalytic oxidation process. **Figure** **5**a shows a schematic of the homemade spectro‐electrochemical cell used for IRAS measurements (see the “Experimental Section” for details). Figure 5b plots the IRAS spectra collected from a branched NiSe_2_ NP‐based electrode in 1 m KOH containing 1 m EG. We observed the asymmetric stretching vibration of O—C—O (*ν*_as_(OCO), 1568 cm^−1^), and —C—H (*δ*(—CH), 1380 cm^−1^), and the symmetric stretching vibration of O—C—O (*ν*_s_(OCO), 1350 cm^−1^), that can be ascribed to the characteristics of HCOO—, formate.^[^ ^34^, ^54^ ^]^ Based on the band at 1568 cm^−1^, oxalate is one of the possible products, since the feature band of oxalate at 1307 cm^−1^ may be submerged by the bands at 1350 cm^−1^. The simultaneous appearance of 1568, 1467, 1350, and 1085 cm^−1^ bands also indicates that glycolate species could be generated, becoming one of the EGOR products.^[^ ^34^ ^]^ Additionally, IRAS spectra showed signs of the presence of carbonate at higher potential, which pointed out that formate was the main product of the EGOR at the NiSe_2_ surface and other carbonates were minor products.

To further examine the EGOR products, a long‐term test was carried out. Figure 5c displays the chronoamperometry (CA) response for these three electrodes at 1.6 V for a continuous 10 h operation in 1 m KOH and 1 m EG. It should be noted that the fluctuation of the curve was associated with the release of oxygen bubbles during the CA operation (Figure S9, Supporting Information). A sustained current density well above 50 mA cm^−2^ was measured for both branched and bundled NiSe_2_‐based electrodes. On the other hand, spherical NPs showed slightly lower current densities at ≈43 mA cm^−2^ after 10 h testing. As it can be seen in Figure S10 (Supporting Information), at an external potential of 1.65 V, after continuous 2 days CA testing, a current density of up to 30 mA cm^−2^ was still measured, higher than the initial value of most of the previously reported catalysts (Table S1, Supporting Information). As shown in Figure S11 (Supporting Information), scanning electron microscopy (SEM)–energy dispersive spectrometer (EDS) analysis of the branched NiSe_2_ electrode after the long‐term CA test demonstrated that the Ni/Se atomic ratio increases from ≈3:7 (Figure S1b, Supporting Information) to 8:2. Besides, the XPS spectra show that the NiSe_2_ was oxidized to Ni(OH)_2_ or NiOOH, with a reduced amount of Se remaining (Figure S12, Supporting Information). The atomic ratio Ni/Se was quantified at ≈9, which is consistent with SEM–EDS data considering just the reconstruction of the material surface. Besides, TEM characterization shows the particle morphology not to change during CA (Figure S13, Supporting Information). The electronic structure and chemical composition change were associated with the surface reconstruction under an external potential in alkaline media.^[^ ^55^, ^56^, ^57^ ^]^

After 10 h CA, a small portion of the electrolyte was analyzed by ^1^H nuclear magnetic resonance (NMR) spectroscopy. As shown in Figure 5d, the NMR spectra confirmed that formate was the main EGOR product. To further determine the reaction products, the electrolyte after long‐term CA was diluted a 20‐fold, and analyzed by ion chromatography (IC). Figure 5e displays the experimental IC curve (black) collected from the branched NiSe_2_‐based electrode and the reference data obtained from 50 mg L^−1^ glycolate (red), formate (blue), and oxalate (pink). The IC curve obtained from the electrolyte displayed peaks at 4.3, 5.2, and 27.6 min, which matched well with the reference peak, revealing the formation of glycolate, formate, and oxalate during EGOR.^[^ ^58^ ^]^ Similar results were obtained from the three different NiSe_2_‐based electrodes (for spherical NP and bundle‐based electrodes; Figure S14, Supporting Information). Quantitatively, based on the standard concentration curve, we determined that around 12.23 mg L^−1^ glycolate, 83.21 mg L^−1^ formate, and 13.93 mg L^−1^ oxalate were produced from the branched NiSe_2_‐based electrode.

The Faradaic efficiency (FE) was calculated using the following equation

(1) $$\mathrm{FE}\;\left(\%\right)=\frac{\mathrm{mol}\;\mathrm{of}\;\mathrm{product}\;\times \;n\;\times \;F}{\mathrm{total}\;\mathrm{charge}\;\mathrm{passed}}\;\times 100\%$$

where *n* is the electron transfer number (4 for glycol to glycolate, 6 for glycol to formate, and 8 for glycol to oxalate) and *F* is the Faradaic constant (96 485 C mol^−1^). As displayed in Figure 5f, the Faradaic efficiency of the glycol‐to‐formate conversion was above 80% for the three electrodes. Besides, the FE values for glycol to glycolate and glycol to oxalate were around 5% and 7%, respectively.

The concept of coupling HER and EGOR in alkaline media was further studied. As shown in Figure S15 (Supporting Information), an H‐cell with two compartments separated by a proton exchange membrane was used to produce hydrogen and quantify its evolution rate. Figure 5g displays the curve of the detected product at the cathode side. As can be seen in Figure 5g, two peaks were located at ≈1.1 and ≈1.5 min, which were associated with molecular hydrogen and argon. By comparing with known standard hydrogen concentrations, the calculated Faradaic efficiency was determined to be nearly 100%, indicating that hydrogen was the only product on the cathode. As shown in Figure S16 (Supporting Information), a sharp rise in the CA curve after adding 1 m EG in the 1 m KOH solution was observed, indicating that the presence of EG significantly promotes the HER at the cathode (Figure 5h).

### 2.4 DFT Calculations

**Figure 6:**
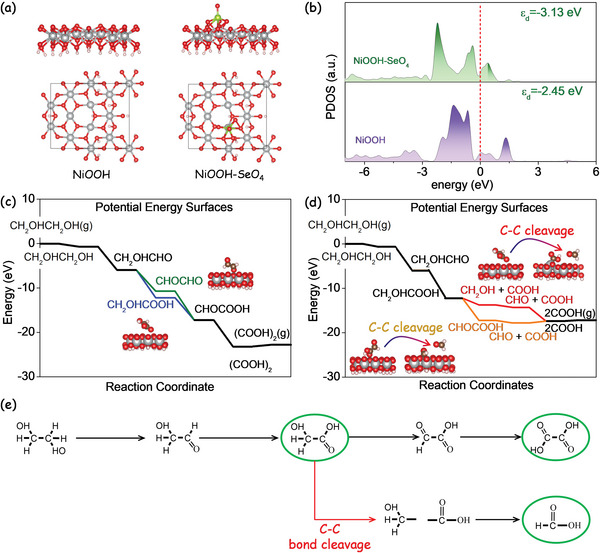
a) Optimized structural model of NiOOH and NiOOH—SeO_4_ from the side and top views. b) PDOS of NiOOH and NiOOH—SeO_4_ regarding the Ni 3d orbitals. c) Gibbs free energy diagrams for the glycol‐to‐oxalate C2 conversion on a NiOOH surface. d) Gibbs free energy diagrams for the glycol‐to‐formate C1 conversion on a NiOOH surface considering the C—C bond cleavage at the glycolate step (red) and the glyoxylate step (yellow). The free energy of each configuration is given in eV. e) Proposed reaction pathway for the electrocatalytic glycol conversion.

To determine the role of Se in the electrocatalytic performance, DFT calculations were used to calculate the partial density of state (PDOS) of the NiOOH–SeO_4_ and the pristine NiOOH structural models (**Figure** **6**a). As shown in Figure 6b, the d electrons of Ni in the SeO_4_‐modified NiOOH were found to significantly concentrate around the Fermi level, leading to an enhancement of the electroconductivity and electron transfer. Thus, the higher EGOR performance of the NiSe_2_‐based electrode could be associated with the presence of SeO*_x_* species during the electrocatalytic process, in agreement with previous publications.^[^ ^42^ ^]^

To gain insight into the reaction mechanism and understand the competition among the dehydrogenation, and C—C bond cleavage at the molecular level, the adsorption energies in the reaction coordinates were determined using DFT calculations. Considering NiOOH as the main active species for EGOR in alkaline solution, we built a slab model of NiOOH (001). For the first step, the glycol binds to the top site via one O atom that was removed from the model, and the calculated binding energy for glycol is −0.67 eV, which is in good agreement with that obtained on the glycol on Pt (211) surface.^[^ ^59^ ^]^

To figure out the reaction pathways for the EGOR in this study, first, we analyzed the pathway for glycol‐to‐oxalate conversion. Figure 6c shows the free energy of adsorbed intermediates for the glycol‐to‐oxalate conversion on the NiOOH surface. According to previous investigations, the adsorbed glycol (CH_2_OHCH_2_OH) requires two dehydrogenations to form the intermediate of glycolaldehyde (CH_2_OHCHO). At this point, the next step involves either further dehydrogenation on the same C and interacting with OH^−^ to form glycolate (CH_2_OHCOOH) or another two dehydrogenations on another C resulting in glycoxal (CHOCHO). As can be seen, both reactions are exothermal, and more energy is released to form glycolate (−6.24 eV) than glycoxal (−4.74 eV). This means that the former reaction pathway of glycolaldehyde to glycolate is favored. With another two dehydrogenations on another C, the glyoxylate (CHOCOOH) is formed. This can finally interact with OH^−^ to produce the oxalate ((COO^−^)_2_).

The glycol‐to‐formate reaction pathway displays more possibilities due to the C—C bond cleavage that occurs on one of the intermediates of the glycol‐to‐oxalate conversion. Figure S15 (Supporting Information) displays the adsorbed intermediates from glycol‐to‐formate conversion on the NiOOH surface regarding the C—C bond cleavage in glycol (CH_2_OHCH_2_OH), glycolaldehyde (CH_2_OHCHO), and glycoxal (CHOCHO). As can be seen in this graph, the reaction pathway for the two dehydrogenations to CH_2_OHCHO is more favored than for the direct C—C bond cleavage in the glycol (CH_2_OHCH_2_OH). Among these three pathways (Figure S17, Supporting Information), the C—C bond cleavage is more likely to occur in the glycoxal (CHOCHO), resulting in two pieces of formaldehyde (CHO). Figure 6d compares the reaction pathway for glycol‐to‐formate conversion that involves the C—C bond cleavage in the glycolate (CH_2_OHCOOH) or the glyoxylate (CHOCOOH). As it can be seen, the C—C bond cleavage is more favored in the former intermediates glycolate (CH_2_OHCOOH). By comparing the C—C bond cleavage in the glycolate (Figure 6d) and glycoxal (Figure S17, Supporting Information), less reaction energy was needed for breaking the C—C bond in glycolate than in glycoxal. Thus, we hypothesize that formate was formed preferentially by the C—C bond cleavage at the glycolate and the posterior further oxidation to formate.

To sum up, the possible EGOR mechanism on a nickel surface in alkaline media is illustrated in Figure 6e. First, NiOOH was formed from the Ni surface reconstruction under the external potential. Then, the glycol was adsorbed on the NiOOH surface, followed by two dehydrogenations on one carbon, and interacting with OH^−^ to form glycolate

(2) $${\mathrm{OH}}^{-}\;+\;\mathrm{Ni}{\left(\mathrm{OH}\right)}_{2}\;\to \;\mathrm{NiOOH}\;+\;H_{2}O\;+\;e$$

(3) $${\mathrm{CH}}_{2}{\mathrm{OHCH}}_{2}{\mathrm{OH}}_{\mathrm{sol}}\;\to \;{\mathrm{CH}}_{2}{\mathrm{OHCH}}_{2}{\mathrm{OH}}_{\mathrm{ads}}$$

(4) $$\begin{array}{ccc} & & {\mathrm{CH}}_{2}\mathrm{OHC}*H_{2}{\mathrm{OH}}_{\mathrm{ads}}\;+\;3OH^{n}\;+\;\mathrm{NiOOH}\\ & & \;\to \;{\mathrm{CH}}_{2}\mathrm{OHC}*HO_{\mathrm{ads}}\;+\;\mathrm{Ni}{\left(\mathrm{OH}\right)}_{2}\;+\;2H_{2}O\;+\;3e \end{array}$$

(5) $$\begin{array}{ccc} & & {\mathrm{CH}}_{2}\mathrm{OHC}*HO_{\mathrm{ads}}\;+\;3OH^{n}\;+\;\mathrm{NiOOH}\\ & & \;\to \;{\mathrm{CH}}_{2}\mathrm{OHC}*\mathrm{OO}H_{\mathrm{ads}}\;+\;\mathrm{Ni}{\left(\mathrm{OH}\right)}_{2}\;+\;2H_{2}O\;+\;3e \end{array}$$

Some of the adsorbed CH_2_OHCOOH was released from the surface and existed in solution in the form of glycolate (CH_2_OHCOO^‐^)

(6) $${\mathrm{CH}}_{2}{\mathrm{OHCOOH}}_{\mathrm{ads}}\;\to \;{\mathrm{CH}}_{2}{\mathrm{OHCOOH}}_{\mathrm{sol}}$$

On the other hand, some of the adsorbed CH_2_OHCOOH undergo two dehydrogenations at another C to form oxalate ((COO^−^)_2_)

(7) $$\begin{array}{ccc} & & C*H_{2}{\mathrm{OHCOOH}}_{\mathrm{ads}}+2OH^{n}+\mathrm{NiOOH}\\ & & \;\to C*\mathrm{HOCOO}H_{\mathrm{ads}}+\mathrm{Ni}{\left(\mathrm{OH}\right)}_{2}+2H_{2}O+2e \end{array}$$

(8) $$\begin{array}{ccc} & & C*\mathrm{HOCOO}H_{\mathrm{ads}}+2OH^{-}+\mathrm{NiOOH}\\ & & \;\to \;{\left(\mathrm{COOH}\right)}_{2\;\;\mathrm{ads}}\;+\;\mathrm{Ni}{\left(\mathrm{OH}\right)}_{2}\;+\;H_{2}O\;+\;2e \end{array}$$

(9) $${\left(\mathrm{COOH}\right)}_{2\mathrm{ads}}\;\to \;{\left(\mathrm{COOH}\right)}_{2\mathrm{sol}}$$

Based on the DFT calculations, the C—C bond cleavage preferentially occurs in the adsorbed CH_2_OHCOOH, forming *CH_2_OH and *COOH, and resulting in the presence of formate in the solution

(10) $${\mathrm{CH}}_{2}{\mathrm{OHCOOH}}_{\mathrm{ads}}+OH^{-}\to *CH_{2}{\mathrm{OH}}_{\mathrm{ads}}+*\mathrm{COO}H_{\mathrm{ads}}$$

The intermediate *COOH interacts with NiOOH in the following way

(11) $$\begin{array}{c} {}*\mathrm{COO}H_{\mathrm{ads}}+\mathrm{NiOOH}+H_{2}O+2e\to {\mathrm{HCOOH}}_{\mathrm{ads}}+\mathrm{Ni}{\left(\mathrm{OH}\right)}_{2}+OH^{-} \end{array}$$

The other intermediate *CH_2_OH probably undergoes two dehydrogenations

(12) $$\begin{array}{c} {}*CH_{2}{\mathrm{OH}}_{\mathrm{ads}}+OH^{-}+\mathrm{NiOOH}\to *\mathrm{CH}O_{\mathrm{ads}}+\mathrm{Ni}{\left(\mathrm{OH}\right)}_{2}+H_{2}O+e \end{array}$$

(13) $$*\mathrm{CH}O_{\mathrm{ads}}+2OH^{-}+\mathrm{NiOOH}\to *\mathrm{HCOO}H_{\mathrm{ads}}+\mathrm{Ni}{\left(\mathrm{OH}\right)}_{2}+2e$$

Finally, the adsorbed formic acid is released from the electrocatalyst surface

(14) $$*\mathrm{HCOO}H_{\mathrm{ads}}\to *\mathrm{HCOO}H_{\mathrm{sol}}$$

## 3 Conclusion

To sum up, we reported on the production of nickel selenide nanostructures with controlled morphology and crystallographic phases. These nanostructures were tested for the EGOR in alkaline electrolyte. The best performance was obtained from branched NiSe_2_ nanoparticles, displaying a current density of up to 95 mA cm^−2^ in an electrolyte containing 1 m KOH and 1 m EG at 1.6 V. Even after 10 h continuous CA operation, the current density was sustained at 56 mA cm^−2^. The improved activity was associated with improved surface coverage, diffusion of active species, and ECSA. Further in situ IRAS and ex situ NMR and IC investigations showed the main product of the EGOR to be formate with up to 80% FE, and with glycolate and oxalate as minor products. DFT calculations provided insights into the oxidation mechanism, demonstrating that the presence of Se promotes the catalyst activity and suggesting that the C—C bond cleavage takes place in the glycolate.

## 4 Experimental Section

### Chemicals

All the chemicals, including selenium powder (200 mesh, 99.5%, Acros Organics), nickel(II) acetylacetonate (Ni(acac)_2_, 96%, Sigma–Aldrich), OAm (80–90%, Acros Organics), OAc (99%, Sigma–Aldrich), KOH (85%, Sigma–Aldrich), potassium carbonate (K_2_CO_3_, 99.5%, Sigma–Aldrich), potassium bicarbonate (KHCO_3_, 99.7%, Sigma–Aldrich), ODE (90%, Sigma‐Aldrich), DDT (98%, Sigma–Aldrich), carbon black (CB, Vulcan XC72, Sigma–Aldrich), EG (99.8%, Sigma Aldrich), and Nafion (10 wt%, perfluorinated ion‐exchange resin, dispersion in water, Sigma–Aldrich), were used as received, without further treatment. Analytical grade chloroform and ethanol for the washing process were obtained from various sources. MilliQ water (18.2 MΩ cm) was used for the preparation of electrolyte and catalytic ink, and IC measurements. An argon‐filled glove box was used to handle sensitive chemicals and reactive ink preparation during the synthesis.

### Synthesis of NiSe_2_

Nanostructured NiSe_2_ was produced from a two‐step process involving the preparation of a reactive ink and its subsequent thermal decomposition. A series of nickel selenide samples, having particles with different morphologies, were produced using the type and ratio of solvents and surfactants as the variable parameter.^[^ ^60^ ^]^ First, the precursor ink was prepared inside an argon‐filled glove box by dissolving 0.4 mm Ni(acac)_2_ and 2.5 mm of Se powder inside a vial with 30 mL OAm and 6 mL DDT under vigorous stirring for 1 h at 60 °C. DDT was essential to dissolve the Se powder. The obtained solution was filtered using a 0.2 µm filter. Then, 10 mL of the prepared precursor together with the appropriate amount of OAm, ODE, and/or OAc was placed in a 50 mL three‐neck flask and vacuumed at 60 °C for 30 min followed by heating to 230 °C under Ar at a rate of 5 °C min^−1^. After reacting for 20 min, the solution was rapidly cooled down to room temperature in a water bath. Then, the crude solution was mixed with 10 mL of chloroform and centrifuged at 8000 rpm for 5 min. This purification process, involving redispersion in chloroform and precipitation using ethanol and centrifugation, was repeated several times. The final product was dissolved in chloroform and stored in the glove box. Different OAm/ODE/OAc ratios were used to produce NiSe_2_ with different geometries and crystal phases: NiSe_2_ quasispherical nanoparticles were obtained using 20 mL of OAm and no ODE; NiSe_2_ branched nanostructures were produced using 5 mL of OAm and 15 mL ODE; NiSe_2_ elongated bundles were obtained in the presence of 5 mL of OAm, 3 mL ODE, and 12 mL OAc.

### Material Characterization

Laboratory XRD was performed on a Bruker AXS D8 Advance (Cu K*α* radiation: *λ* = 1.5106 Å). SEM together with an EDS was performed on a Gemini 300 field emission scanning electron microscope (ZEISS, Germany) equipped with an AZtecOne UltimMax40 energy spectrometer. HRTEM and STEM investigations were performed on a field emission gun FEI Tecnai F20 microscope. HAADF–STEM was combined with EELS in the Tecnai microscope using a GATAN QUANTUM filter.^[^ ^61^, ^62^ ^]^ XPS analyses were conducted on a SPECS system.

### Electrochemical Characterization

Electrochemical characterization was performed at room temperature on a CorrTest potentiostat. A conventional three‐electrode cell was used, containing a Pt wire as a counter electrode (CE), Hg/HgO as a reference electrode (RE), and GC (5 mm diameter) as the working electrode (WE). To prepare the catalytic ink, 5 mg of dried particles and 10 mg of CB were dispersed in a vial containing 1 mL of MilliQ water and 1 mL of ethanol and 100 µL of 10 wt% Nafion, followed by half an hour vigorous sonication. Then, 5 µL of the prepared ink was drop‐casted on the polished GC electrode and dried naturally in the open air. Conventional electrochemical techniques including CV and CA were applied to study the activities and stabilities. Current densities were normalized to the geometrical area (0.196 cm^−2^) of the GC electrode. The measured potentials (vs Hg/HgO) were converted to the RHE according to the equation *E*_RHE_ = *E*_Hg/HgO_ + 0.059 × pH + *E* ^ϴ^_Hg/HgO_ (where *E*_Hg/HgO_ is the measured potential, *E* ^ϴ^_Hg/HgO_ is the reference potential of 0.098 V according to the manufacturer guide, and pH is the practical value of 13.6 for 1.0 m KOH). Electrochemical impedance spectra were obtained in 1 m KOH in the presence of 1 m EG from 10^−1^ to 10^5^ Hz. To identify and quantify the reaction products, after long‐term CA, the electrolyte was characterized by ^1^H NMR spectroscopy and IC. For IC measurement, a freshly prepared 4.5 mm K_2_CO_3_ and 0.8 mm KHCO_3_ solution was used as leachate solution. At the cathode, the generated hydrogen was determined by gas chromatography (GC, 8890, Agilent Technologies) connected to the electrocatalytic cell. Before measurement, a stable flow rate of 20 cm^3^ min^−1^ Ar (99.999%) was bubbled for 30 min at the Pt wire in an H‐type two‐compartment cell, which was separated by a Nafion‐115 proton membrane. The GC product analysis was conducted every 12 min. The produced gas was identified and quantified by a calibration curve using known concentrations of standard H_2_ gas.

### In Situ Electrochemical IRAS

In situ IRAS measurements were performed using a homemade spectro‐electrochemical cell. The incident angle of the IR beam was set at 55°. Before spectra gathering, a ≈10 µm thick layer containing 1 m KOH and 1 m EG electrolyte was generated by pressing the WE onto the CaF_2_ prism surface, allowing the infrared radiation to travel through the CaF_2_ prism and reflect at the WE into the mercury–cadmium telluride (MCT) detector. The infrared spectrum was collected every 5 s, and potential scanning was 5 mV s^−1^. Thus the spectrum was generated by the average co‐adding of ≈44 interferograms. All the spectra in this work were shown in the absorbance units defined as −log(*I*/*I*_0_), where *I* and *I*_0_ represent the absorption intensities at the sample and correlative conditions, respectively.

### Computational Method

The Vienna ab initio package (VASP) was used to perform all the DFT calculations within the generalized gradient approximation (GGA) using the Perdew, Burke, and Ernzerhof (PBE) formulation.^[^ ^63^, ^64^, ^65^ ^]^ The projected augmented wave (PAW) potential was used to describe that the ionic cores and valence electrons were taken into account using a plane wave basis set with a kinetic energy cutoff of 400 eV.^[^ ^66^, ^67^ ^]^ Partial occupancies of the Kohn–Sham orbitals were allowed using the Gaussian smearing method and a width of 0.05 eV. The electronic energy was considered self‐consistent when the energy change was smaller than 10^−5^ eV. Geometry optimization was considered convergent when the force change was smaller than 0.02 eV Å^−1^. Grimme's DFT‐D3 methodology was used to describe the dispersion interactions.^[^ ^68^ ^]^

Using a 7×11×9 Monkhorst–Pack *k*‐point grid for Brillouin zone sampling, the equilibrium lattice constants of the monoclinic NiOOH unit cell were optimized to be *a* = 5.168 Å, *b* = 2.847 Å, *c* = 4.516 Å, at *α* = 90°, *β* = 107.1°, and *γ* = 90°. They were used to construct a NiOOH(001) surface model with *p*(2×3) periodicity in the *x*‐ and *y*‐directions and one stoichiometric layer in the *z*‐direction separated by a vacuum layer in the depth of 15 Å to separate the surface slab from its periodic duplicates. One O atom was removed from the model to create an oxygen vacancy. During structural optimizations, the gamma point in the Brillouin zone was used for *k*‐point sampling, and all atoms were allowed to relax.

The free energy of a gas phase molecule or an adsorbate on the surface was calculated by the equation *G* = *E* + ZPE − TS, where *E* is the total energy, ZPE is the zero‐point energy, *T* is the temperature in kelvin (298.15 K), and *S* is the entropy.

## Conflict of Interest

The authors declare no conflict of interest.

## Supporting information

Supporting Information

## Data Availability

The data that support the findings of this study are available from the corresponding author upon reasonable request.
