# Supplementary material for: Selective Ethylene Glycol Oxidation to Formate on Nickel Selenide with Simultaneous Evolution of Hydrogen
Source: Adv Sci (Weinh). 2023 Mar 22;10(15):2300841. doi: 10.1002/advs.202300841 (PMC10214232; doi:10.1002/advs.202300841)
Supplement: Supplementary file 1 — Supporting Information [file ADVS-10-2300841-s001.pdf]

ADVANCED SCIENCE
Open Access

Supporting Information

for *Adv. Sci.*, DOI 10.1002/advs.202300841

Selective Ethylene Glycol Oxidation to Formate on Nickel Selenide with Simultaneous Evolution of Hydrogen

Junshan Li, Luming Li, Xingyu Ma, Xu Han, Congcong Xing, Xueqiang Qi, Ren He, Jordi Arbiol, Huiyan Pan, Jun Zhao, Jie Deng, Yu Zhang, Yaoyue Yang* and Andreu Cabot**

Supporting Information

Selective Ethylene Glycol Oxidation to Formate on Nickel Selenide with Simultaneous Evolution of Hydrogen

Junshan Li,[a] Luming Li,[a,b] Xingyu Ma,[c] Xu Han,[d] Congcong Xing,[e] Xueqiang Qi,[e] Ren He,[e] Jordi Arbiol,[d,f] Huiyan Pan,[g] Jun Zhao,[h] Jie Deng,[b] Yu Zhang,[e,i]* Yaoyue Yang,[c]* Andreu Cabot[e,f]*

a Institute for Advanced Study, Chengdu University, 610106, Chengdu, China

b College of Food and Biological Engineering, Chengdu University, Chengdu 610106, China

c School of Chemistry and Environment, Southwest Minzu University, 610041, Chengdu, China

d Catalan Institute of Nanoscience and Nanotechnology (ICN2), CSIC and BIST, Campus UAB, Bellaterra, 08193 Barcelona, Catalonia, Spain.

e Catalonia Institute for Energy Research - IREC, Sant Adrià de Besòs, Barcelona, 08930, Catalonia, Spain.

f ICREA, Pg. Lluís Companys 23, 08010 Barcelona, Catalonia, Spain.

g School of Biological and Chemical Engineering, Nanyang Institute of Science and Technology, Nanyang 473004, China

h Hebei Key Laboratory of Photoelectric Control on Surface and Interface, College of Science, Hebei University of Science and Technology, Shijiazhuang 050018, China

i Department of Materials Science and Engineering, Pennsylvania State University, University Park, Pennsylvania 16802, United States

SEM-EDS characterization

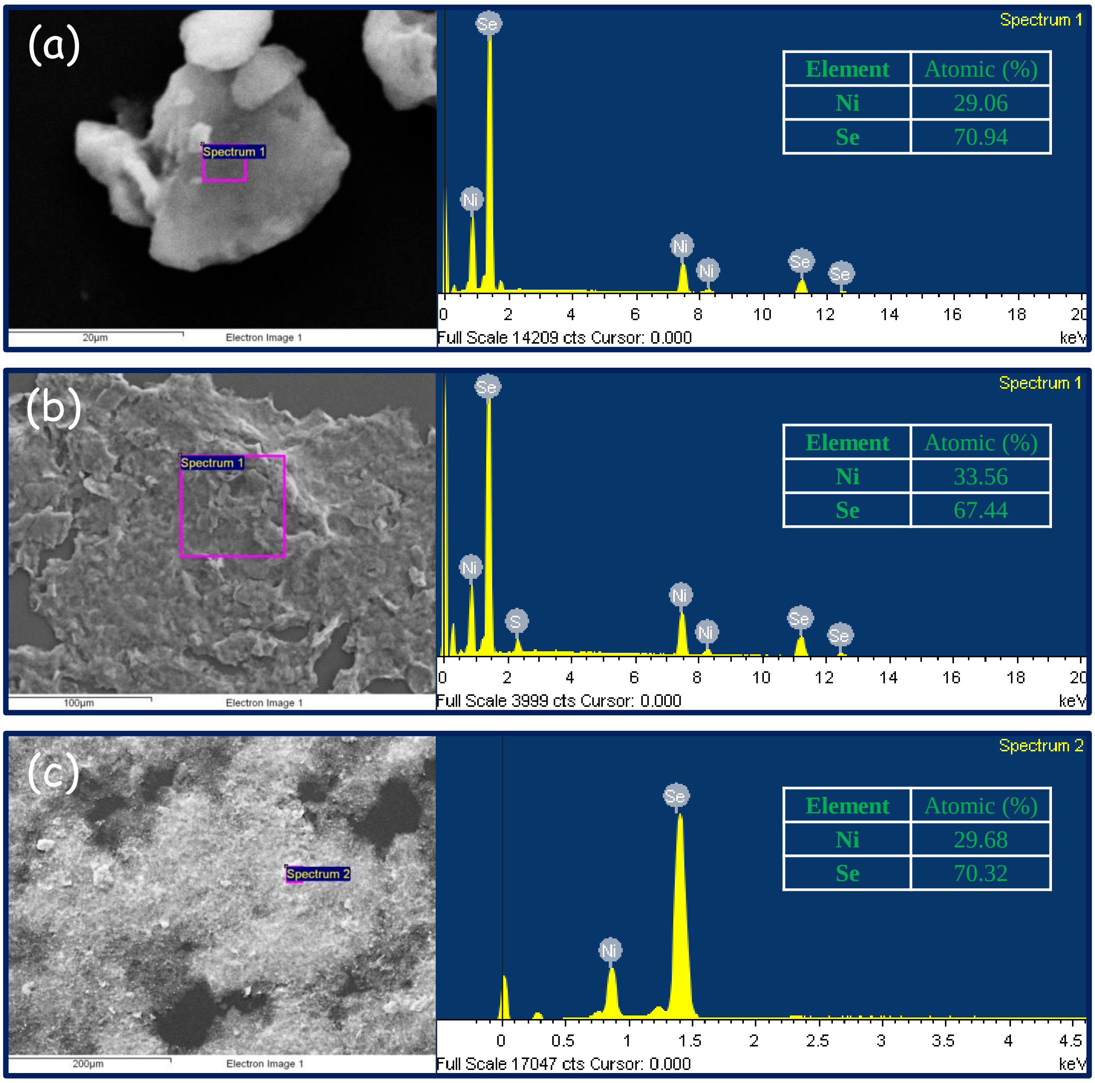

Figure S1. SEM-EDS measurement for the $NiSe_2$ samples with spherical (a), bundle (b), and branched (c) morphology.

HRTEM characterization

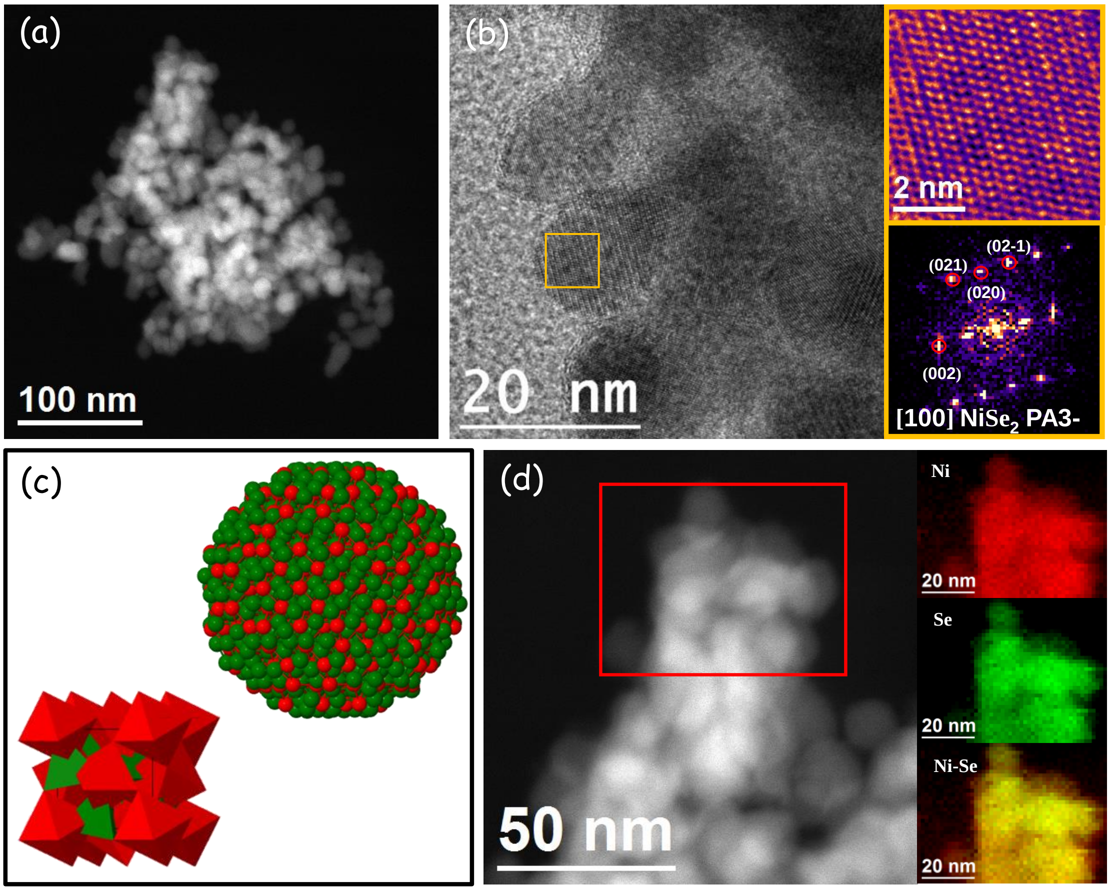

Figure S2. (a) HAADF-STEM micrographs of spherical $NiSe_2$ NPs. (b) HRTEM micrograph, detail of the orange squared region and its corresponding power spectrum. (c) 1*1*1 unit crystal model of $NiSe_2$ and atomic supercell model illustration of the $NiSe_2$ NRs. Red and green particles represent Ni and Se, respectively. (d) EELS chemical composition maps obtained from the red squared area of the STEM micrograph. Individual Ni $N_{2,3}$-edges at 855 eV (red), Se M_1-edges at 231 eV (green) and composites of Ni-Se.

XPS spectra

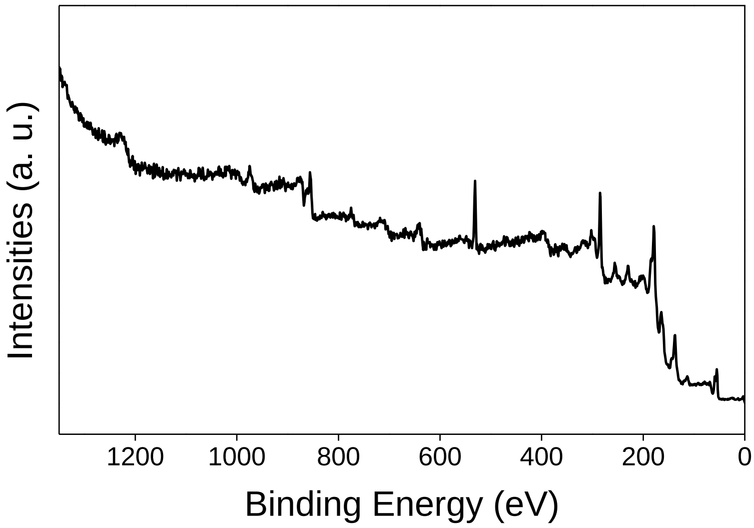

Figure S3. Survey XPS spectrum for the branched nickel selenide.

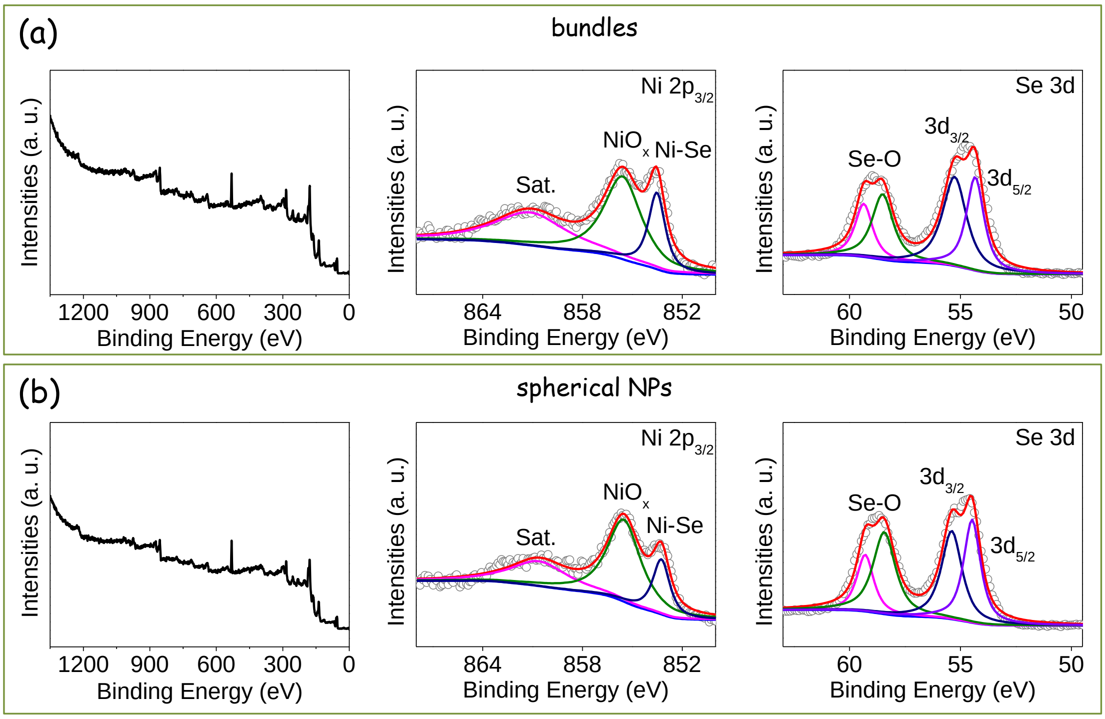

Figure S4. Survey XPS spectrum for the bundles (a) and spherical (b) nickel selenide.

Electrochemical characterization

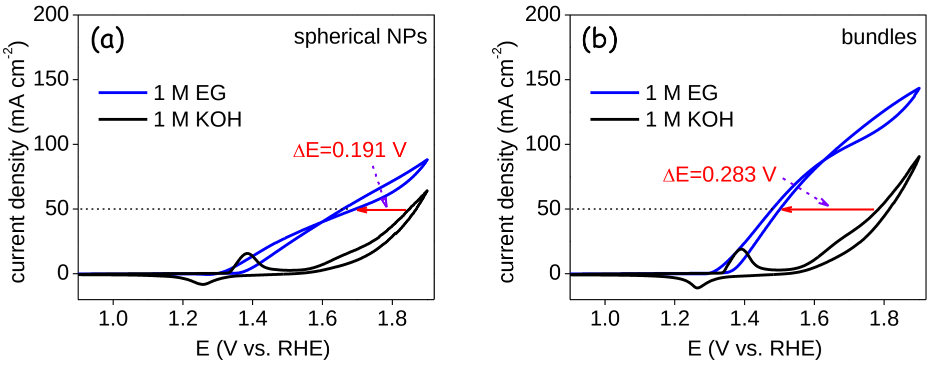

Figure S5. CV curve of (a) spherical NPs and (b) bundles $NiSe_2$ based-electrode in 1 M KOH with and without 1 M EG in the potential range of 0.9-1.9 V vs. RHE.

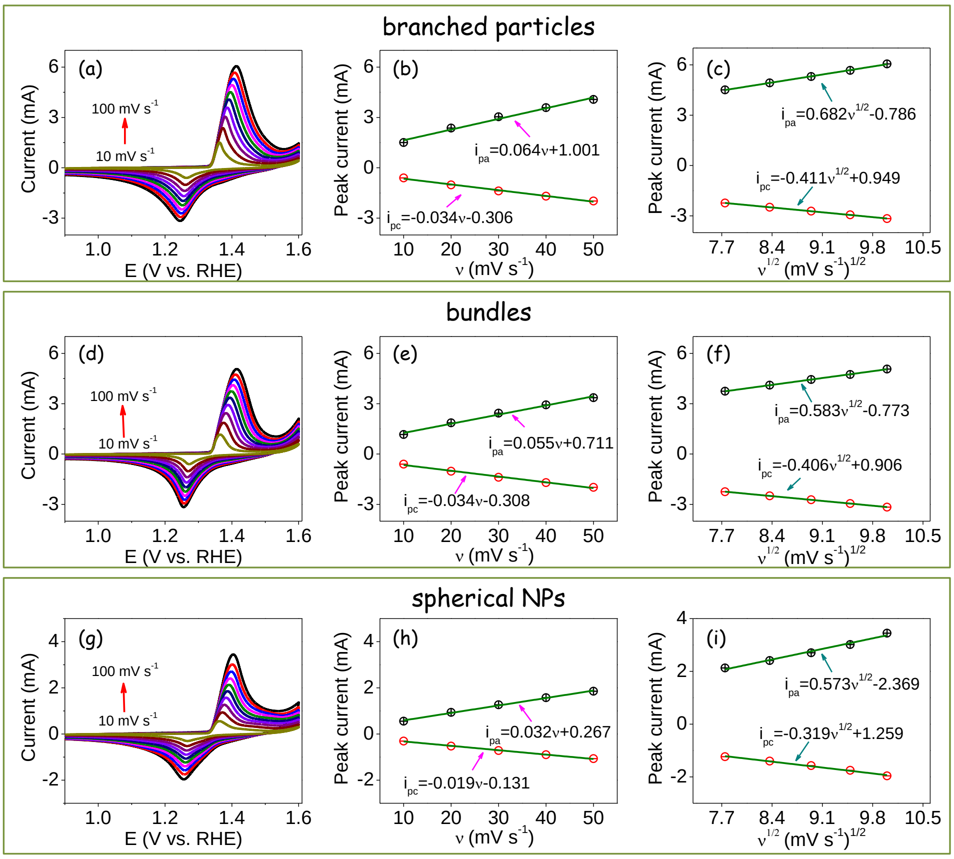

Figure S6. Surface coverage of redox species (Γ*) and diffusion coefficient (D) calculation in 1.0 M KOH for the nickel selenide electrodes displayed on the top of the graph. (a,d,g) CVs with scanning rate from 10 to 150 mV s^{-1} in alkaline media. (b,e,h). Linear fitting of the anodic and cathodic peak current with the scanning rate. (c,f,i) Linear fitting of the anodic and cathodic peak current with the square root of scanning rate.

As shown in Fig. S6adg, when increasing the sweep rate, the position of the anodic peak shifted to higher potential values and the cathodic peak moved to lower potentials for the these three electrode in 1.0 M KOH. The current of both anodic and cathodic peaks rose linearly with increasing scan rate (Fig. S6beh). From the average slope of the anodic and cathodic peaks *vs.* v, the surface coverage of redox species (Γ^*) was estimated:[1]

$$I_p = \left(\frac{n^2F^2}{4RT}\right)A\Gamma^* v$$

where n, F, R, T and A are the number of transferred electrons (assumed to be 1), the Faraday constant (96845 C mol^{-1}), the gas constant (8.314 J K^{-1} mol^{-1}), temperature and the glassy carbon electrodes surface area (0.196 cm^2), respectively.

From this equation, the surface coverage of redox species of starlike $NiSe_2$ electrode to be 2.66×10^{-7} mol cm^{-2}, decreased with the dimensions, to 2.42×10^{-7} mol cm^{-2} for nanobundles and 1.38×10^{-7} mol cm^{-2} for NPs nanostructures.

Also, a linear relationship could be fitted to the dependence of the peak current density with the square root of the voltage scanning rate for the these three electrode in 1 M KOH (Figure S6cfi). This dependence is generally related with a diffusion-limited $Ni(OH)_2 \leftrightarrow$ NiOOH redox reaction, where the proton diffusion within the particle is considered the diffusion process that limits the reaction rate: [1]

$$I_p = 2.69 \times 10^5 n^{3/2} A D^{1/2} C v^{1/2}$$

Where I_p is the peak current, n is the number of transferred electron, A is the geometric surface area of the GC, D is the diffusion coefficient, C is the proton concentration and was estimated to be 3.97 g cm^{-3},[2] we estimated at 0.043 mol cm^{-3}, and υ is the potential scan rate respectively.

The diffusion coefficients in 1 M KOH concentrations were estimated using the above equations, with 1.49×10^{-9} cm^2 s^{-1} , 1.83×10^{-9} cm^2 s^{-1}, and 2.23×10^{-9} cm^2 s^{-1} for NPs, nanobundles, and star-like nickel diselenide, respectively.

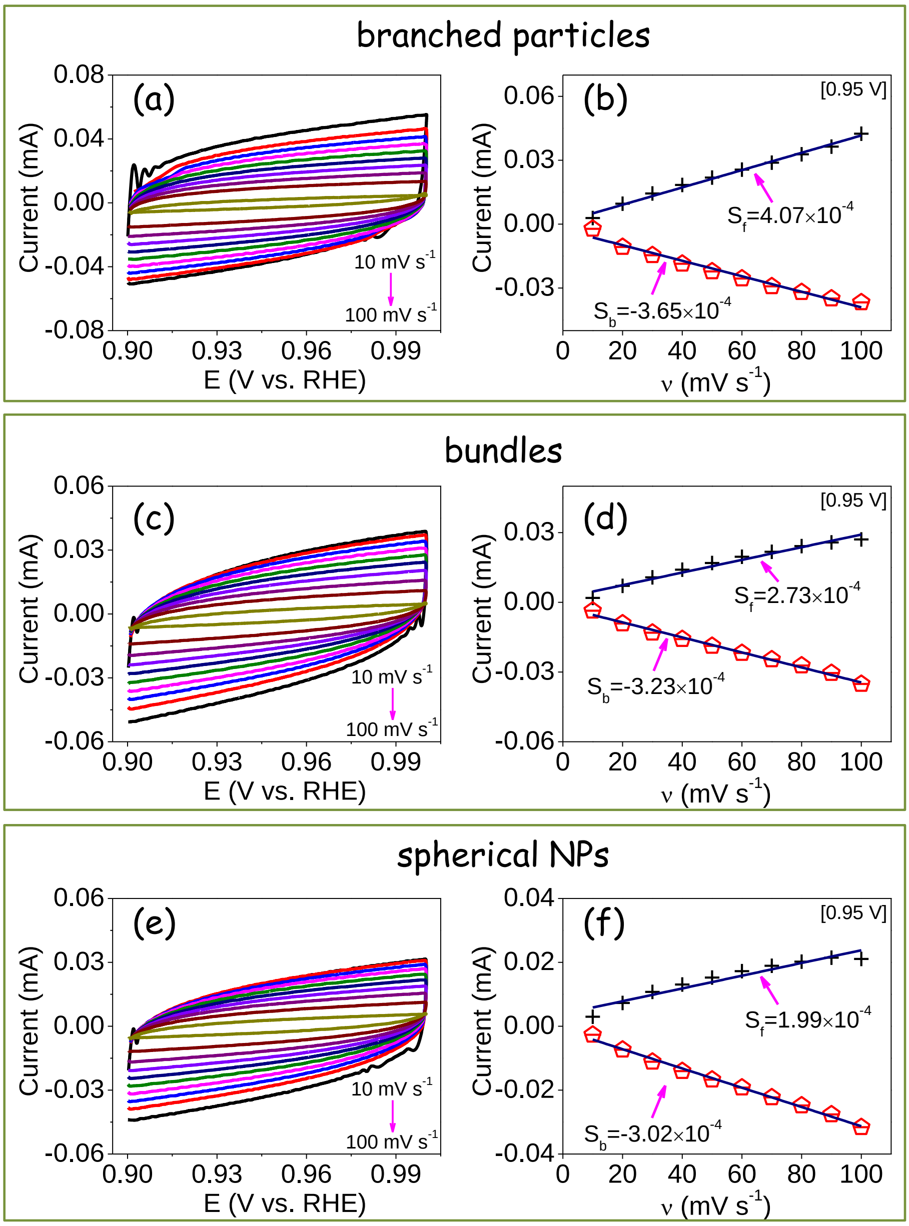

Figure S7. ECSA curves in 1 M KOH for the electrodes displayed on the top of the graph. (a,c,e) CV curves with scanning rate from 10 to 100 mV s^{-1}. (b,d,f) Corresponding linear fitting between current and scan rate.

Electrochemically active surface areas (ECSA) were estimated from the electrochemical double-layer capacitance (C_{dl}) on the basis of CVs recorded at different scan rates in the non-faradaic potential range 0.9-1.0 V vs. RHE (Figure S6ace).[3] Plotting the capacitive current (I) vs. the scan rate (v) yielded a straight line with a slope equal to C_{dl} (Figure S7bdf). ECSA was calculated by dividing C_{dl} by the specific capacitance (C_s):[4]

$$ECSA = C_{dl}/C_s$$

where C_s is 0.04 mF cm^{-2} based on values reported for metal electrodes in aqueous NaOH solution.[5]

Still, using the above equation, the ECSA values were determined to be 5.3 cm^{-2} for NPs and 6.3 cm^{-2} for nanobundels, to 9.65 cm^{-2} for star-like nanostructures, respectively.

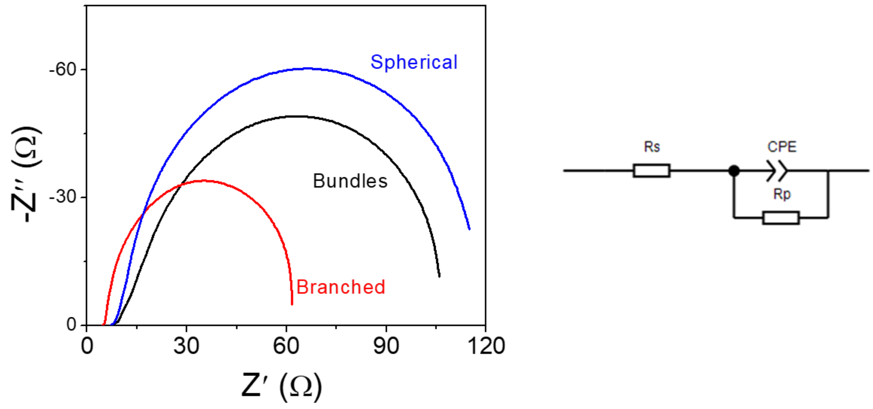

$NiSe_2$ particle morphology	1 M KOH + 1 M EG at 1.5 V vs. RHE	
	R_s (ohm)	R_p (ohm)
Nanobundles	9.1	75.5
Star-like particles	5.1	37.2
Spherical	8.1	83.4

Figure S8. Nyiquist plot of the EIS spectra of the different electrocatalysts in 1 M KOH + 1M EG solution at 1.5 V vs. RHE, fitted circuit and fitting results.

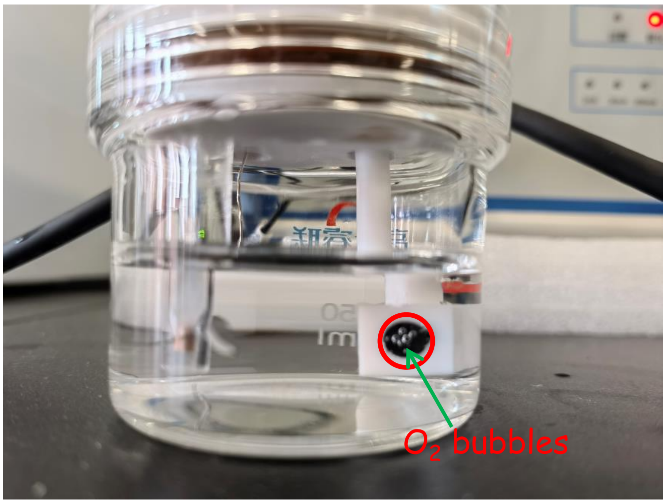

Figure S9. Oxygen bubbles generated at the anode during the long-term CA test in 1 M KOH with 1 M EG.

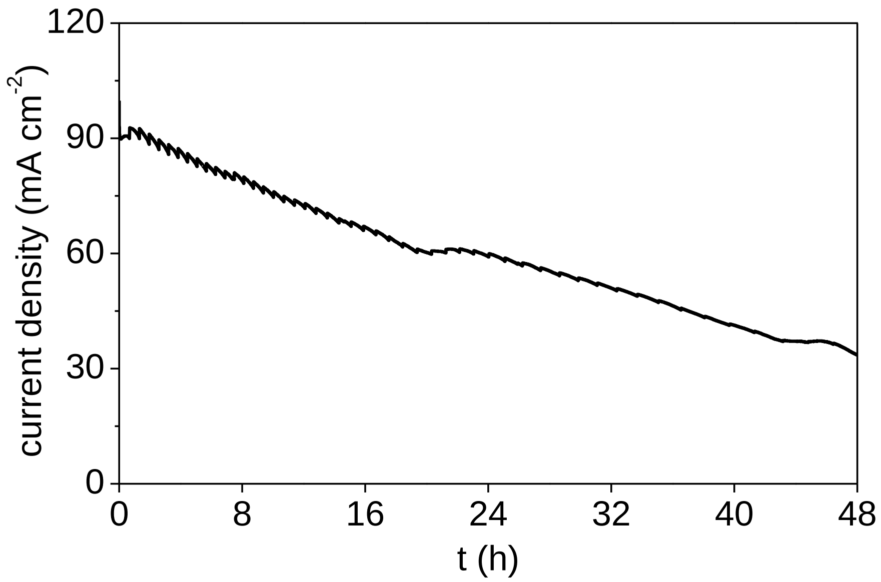

Figure S10. CA response for the branched particles based electrodes at 1.65 V with continuous 48 h operation in 1 M KOH and 1 M EG.

EGOR Electrocatalytic performance comparision with previous results

Table S1. Comparison of the EG oxidation performance between this work and previously reported electrocatalysts with a Ni-based noble-metal-free system.

Catalyst	Synthesis method	Electrolyte	Activity mA cm^{-2}@RHE	Stability Decay(%)@time	Main product and FE	Ref.
FeCoNi/C	precipitation & annealing	1 M KOH + 1 M EG	n.a.	n.a.@~2h	glycolate (36.0%), oxalate (43.5%)	6
Ni NPs/ITO	implantation	0.2 M NaOH + 0.03 M EG	~1.45mA@1.6V	~21%@0.83h	oxalate (n.a.)	7
Ni/carbon paste	electrodeposition	0.1 M NaOH + 0.25 M EG	~0.9mA@1.6V	n.a.	n.a.	8
rGO-NiMn	microwave radiation	1 M KOH + 1 M EG	~47.5@1.5V	~26.3%@2h	n.a.	9
Ni/WC NPs@C	solution & annealing	1 M KOH + 1 M EG	~68@1.6V	n.a.	n.a.	10
Ni-Co Oxides	electrodeposition	1 M KOH + 1 M EG	~55@1.5V	n.a.	oxalate (n.a.)	11
Fe–Co–Ni/C	solution & annealing	0.5 M KOH + 1 M EG	~38.5@1.5V	~25%@1h	n.a.	12
$Ni(OH)_2$-NZB-MW/ CPE	hydrothermal	1.6 M NaOH + 0.14 M EG	~14.0@1.6V	~35.7%@0.28h	n.a.	13
FeCoNi/C	precipitation & annealing	1 M KOH + 1 M EG	~18@1.2V	~95%@0.14h	glycolate (~40%), oxalate (~40%)	14
Ni/Cu/C	electrodeposition	0.1 M KOH + 0.1 M EG	~0.055mA@1.6V	n.a.	n.a.	15
**$NiSe_2$ NPs/C**	**ink & thermal treatment**	**1 M KOH + 1 M EG**	**61.6@1.6V**	**30.7%@10h**	**formate (81.6%), oxalate (4.7%), glycolate (9.3%)**	This work
**$NiSe_2$ bundles/C**	**ink & thermal treatment**	**1 M KOH + 1 M EG**	**81.8@1.6V**	**31.7%@10h**	**formate (83.5%), oxalate (4.1%), glycolate (5.6%)**	This work
**Branched $NiSe_2$ /C**	**ink & thermal treatment**	**1 M KOH + 1 M EG**	**103.6@1.6V**	**45.4%@10h**	**formate (83.4%), oxalate (5.4%), glycolate (7.4%)**	This work

Note: For comparison, the applied potential was intended to convert to be vs. RHE using the following equation:

$$E_{RHE} = E^0_{Ref} + E_{Ref} + 0.059 \times PH$$

Where E^0_{Ref} is potential of the reference, E_{Ref} is the potential that measured *vs.* reference; PH is simply converted from the alkaline electrolyte.

n.a. is the abbreviation of “not available” form the published manuscript.

Sample characterization after CA operation

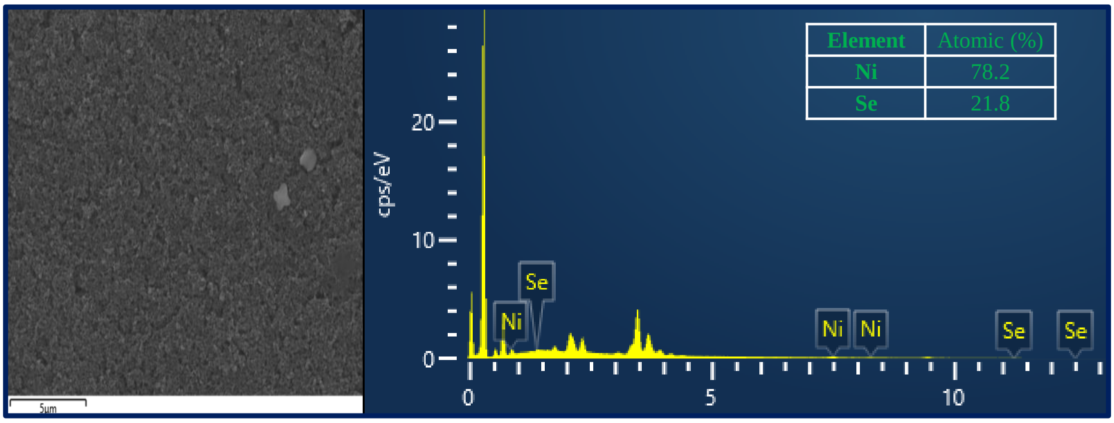

Figure S11. SEM-EDS measurement from branched nickel selenide after 10h CA EGOR operation.

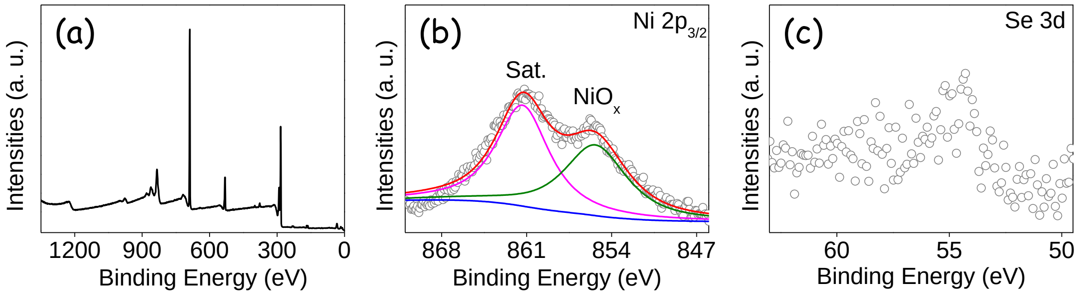

Figure S12. XPS spectra from branched nickel selenide after 10h CA EGOR operation. (a) survey, (b) Ni $2p_{3/2}$, and (c) Se 3d.

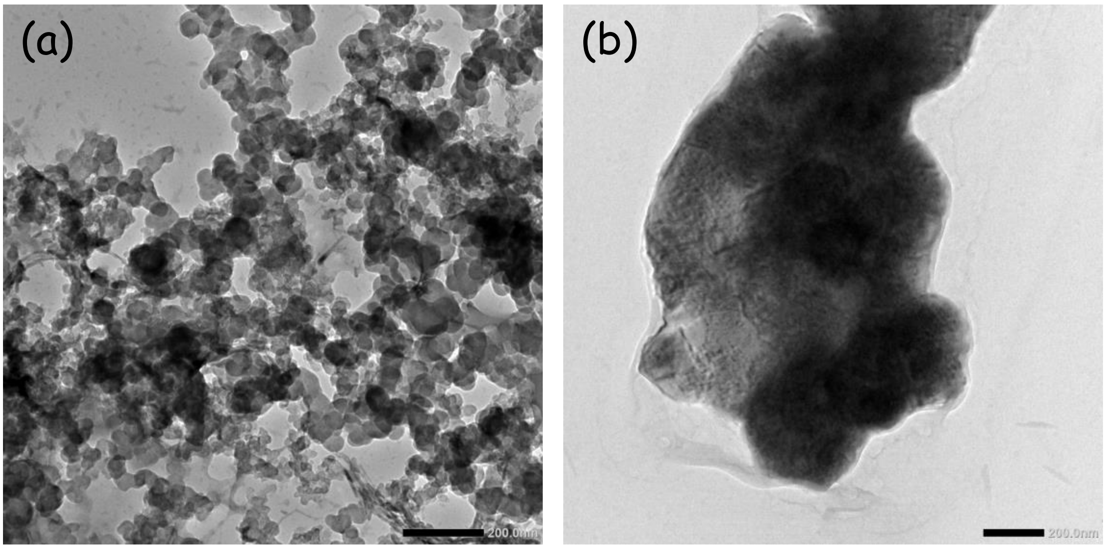

Figure S13. TEM characterization of branched nickel selenide after 10h CA EGOR operation.

IC Profile

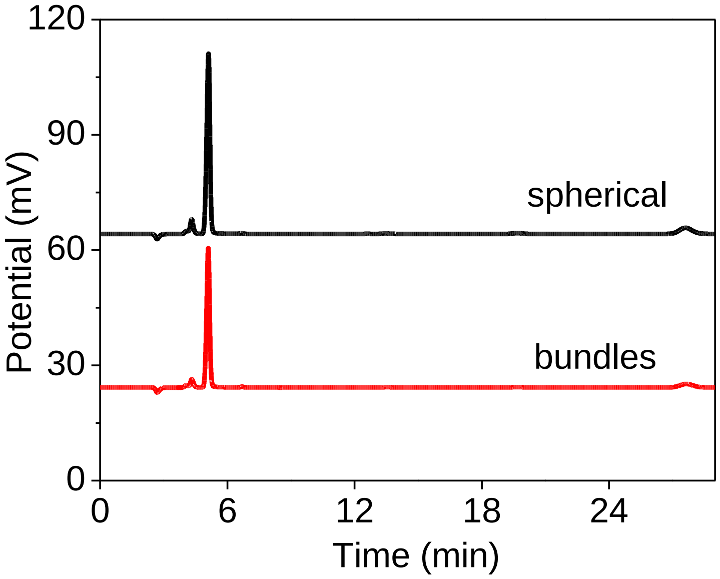

Figure S14. *Ex situ* IC profiles for the electrolyte after CA measurement of elecrtrodes based on spherical and bundles nickel selenide.

Electrolytic cell coupling HER and EGOR

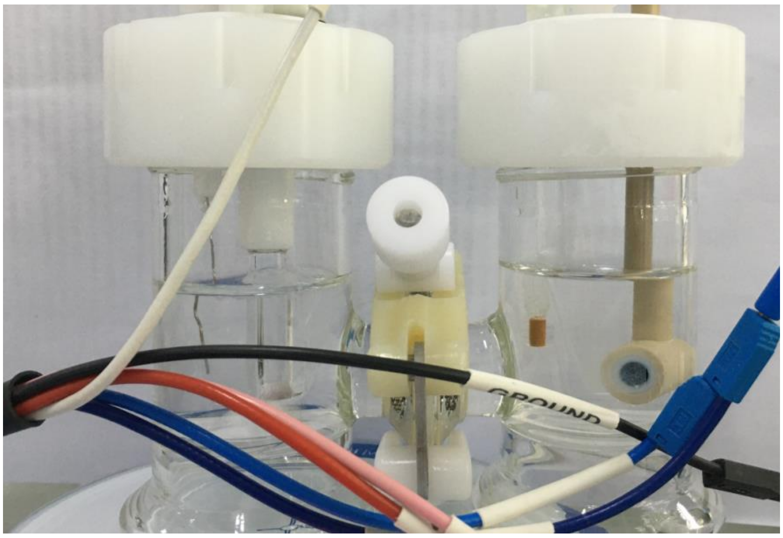

Figure S15. Setup for the H-type cell with two compartments.

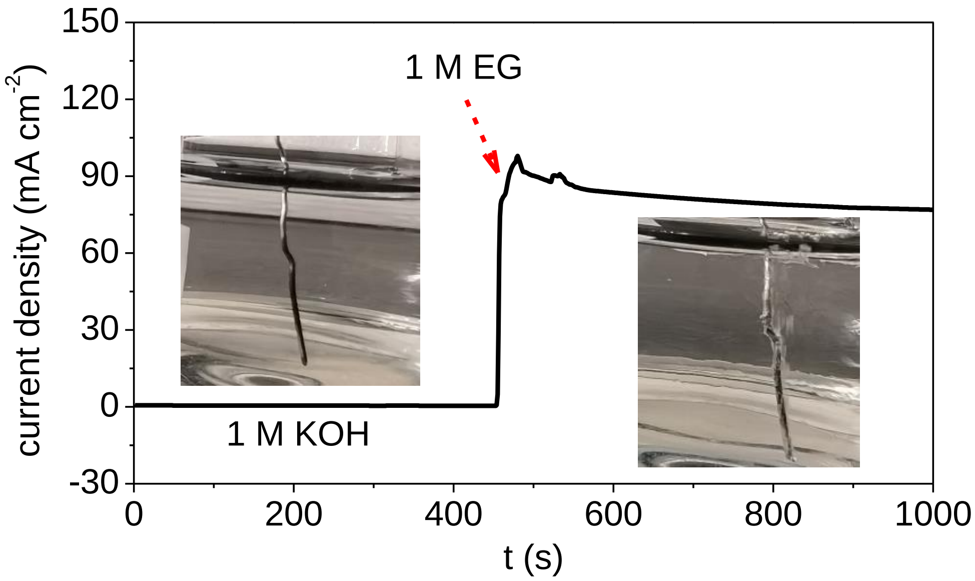

Figure S16. Cathode HER reactions comparision before and after adding 1 M EG into 1 M KOH solution during the CA measurement at 1.6 V vs. RHE.

DFT data

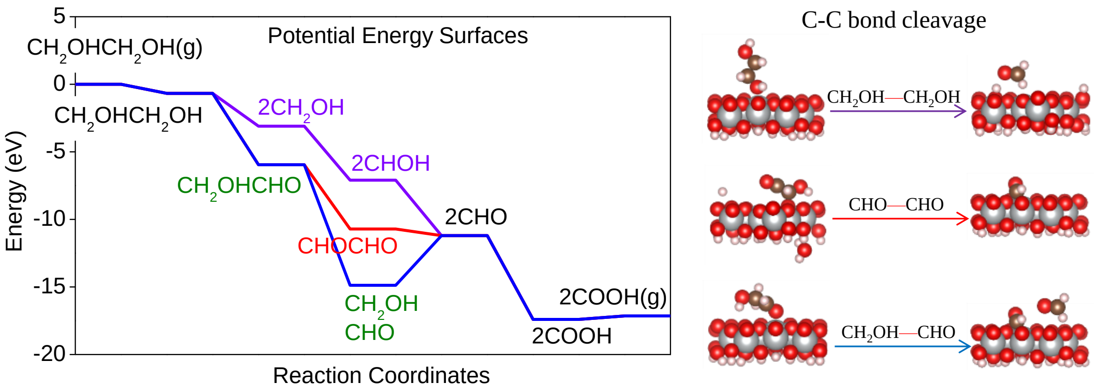

Figure S17. Gibbs free energy diagrams for the glycol-to-formate conversion on NiOOH surface.

Reference

(1) Bard, A. J.; Faulkner, L. R. *Electrochemical Methods: Fundamentals and Applications*, 2nd ed.; 2001.

(2) Zhang, S. J.; Zheng, Y. X.; Yuan, L. S.; Zhao, L. H. Ni-B Amorphous Alloy Nanoparticles Modified Nanoporous Cu toward Ethanol Oxidation in Alkaline Medium. *J. Power Sources* **2014**, *247*, 428–436.

(3) Benck, J. D.; Chen, Z.; Kuritzky, L. Y.; Forman, A. J.; Jaramillo, T. F. Amorphous Molybdenum Sulfide Catalysts for Electrochemical Hydrogen Production: Insights into the Origin of Their Catalytic Activity. *ACS Catal.* **2012**, *2* (9), 1916–1923.

(4) McCrory, C. C. L.; Jung, S.; Ferrer, I. M.; Chatman, S. M.; Peters, J. C.; Jaramillo, T. F. Benchmarking Hydrogen Evolving Reaction and Oxygen Evolving Reaction Electrocatalysts for Solar Water Splitting Devices. *J. Am. Chem. Soc.* **2015**, *137* (13), 4347–4357.

(5) McCrory, C. C. L.; Jung, S.; Peters, J. C.; Jaramillo, T. F. Benchmarking Heterogeneous Electrocatalysts for the Oxygen Evolution Reaction. *J. Am. Chem. Soc.* **2013**, *135* (45), 16977–16987.

(6) Matsumoto, T.; Sadakiyo, M.; Ooi, M. L.; Yamamoto, T.; Matsumura, S.; Kato, K.; Takeguchi, T.; Ozawa, N.; Kubo, M.; Yamauchi, M. Atomically Mixed Fe-Group Nanoalloys: Catalyst Design for the Selective Electrooxidation of Ethylene Glycol to Oxalic Acid. *Phys. Chem. Chem. Phys.* **2015**, *17* (17), 11359–11366.

(7) Lin, Q.; Wei, Y.; Liu, W.; Yu, Y.; Hu, J. Electrocatalytic Oxidation of Ethylene Glycol and Glycerol on Nickel Ion Implanted-Modified Indium Tin Oxide

Electrode. *Int. J. Hydrogen Energy* **2017**, *42* (2), 1403–1411.
(8) Ojani, R.; Raoof, J. B.; Rahemi, V. Evaluation of Sodium Dodecyl Sulfate Effect on Electrocatalytic Properties of Poly (4-Aminoacetanilide)/Nickel Modified Carbon Paste Electrode as an Efficient Electrode toward Oxidation of Ethylene Glycol. *Int. J. Hydrogen Energy* **2011**, *36* (20), 13288–13294.
(9) Hefnawy, M. A.; Fadlallah, S. A.; El-Sherif, R. M.; Medany, S. S. Nickel-Manganese Double Hydroxide Mixed with Reduced Graphene Oxide Electrocatalyst for Efficient Ethylene Glycol Electrooxidation and Hydrogen Evolution Reaction. *Synth. Met.* **2021**, *282*, 116959.
(10) Zhang, M.; Zhu, J.; Wan, R.; Liu, B.; Zhang, D.; Zhang, C.; Wang, J.; Niu, J. Synergistic Effect of Nickel Oxyhydroxide and Tungsten Carbide in Electrocatalytic Alcohol Oxidation. *Chem. Mater.* **2022**, *34* (3), 959–969.
(11) Sun, S.; Zhou, Y.; Hu, B.; Zhang, Q.; Xu, Z. J. Ethylene Glycol and Ethanol Oxidation on Spinel Ni-Co Oxides in Alkaline. *J. Electrochem. Soc.* **2016**, *163* (2), H99–H104.
(12) Gayathri, A.; Kiruthika, S.; Selvarani, V.; AlSalhi, M. S.; Devanesan, S.; Kim, W.; Muthukumaran, B. Evaluation of Iron-Based Alloy Nanocatalysts for the Electrooxidation of Ethylene Glycol in Membraneless Fuel Cells. *Fuel* **2022**, *321*, 124059.
(13) Eshagh-Nimvari, S.; Karim Hassaninejad-Darzi, S. Synergistic Effects of Nanozeolite Beta-MWCNTs on the Electrocatalytic Oxidation of Ethylene Glycol: Experimental Design by Response Surface Methodology. *Mater. Sci. Eng. B Solid-State Mater. Adv. Technol.* **2021**, *268*, 115125.
(14) Matsumoto, T.; Sadakiyo, M.; Ooi, M. L.; Kitano, S.; Yamamoto, T.; Matsumura, S.; Kato, K.; Takeguchi, T.; Yamauchi, M. CO2-Free Power Generation on an Iron Group Nanoalloy Catalyst via Selective Oxidation of Ethylene Glycol to Oxalic Acid in Alkaline Media. *Sci. Rep.* **2014**, *4* (1), 1–6.
(15) Zhou, Y.; Shen, Y.; Li, H. Effects of Metallic Impurities in Alkaline Electrolytes on Electro-Oxidation of Water and Alcohol Molecules. *J. Electrochem. Soc.* **2021**, *168* (12), 124516.
